# Treatment of Fusarium Infection of the Central Nervous System: A Review of Past Cases to Guide Therapy for the Ongoing 2023 Outbreak in the United States and Mexico

**DOI:** 10.1007/s11046-023-00790-6

**Published:** 2023-08-31

**Authors:** Martin Hoenigl, Jeffrey D. Jenks, Matthias Egger, Marcio Nucci, George R. Thompson

**Affiliations:** 1 Division of Infectious Diseases, Department of Internal Medicine, ECMM Excellence Center for Medical Mycology, Medical University of Graz, Auenbruggerplatz 15, 8036 Graz, Austria; 2 BioTechMed, Graz, Austria; 3 Durham County Department of Public Health, Durham, NC USA; 4 Division of Infectious Diseases, Department of Medicine, Duke University, Durham, NC USA; 5 Universidade Federal do Rio de Janeiro (UFRJ), Rio de Janeiro, RJ Brazil; 6 Grupo Oncoclinicas, Rio de Janeiro, RJ Brazil; 7 University of California Davis Center for Valley Fever, Davis, CA USA; 8 Division of Infectious Diseases, Department of Internal Medicine, University of California Davis Medical Center, University of California-Davis Health, 1450 V Street, Suite G500, Sacramento, CA USA; 9 Department of Medical Microbiology and Immunology, University of California Davis, Davis, CA USA

**Keywords:** Fusariosis meningitis, *Fusarium solani*, *Fusarium* spp., Fungal meningitis, Fungal meningitis outbreak

## Abstract

**Introduction:**

Fusariosis of the central nervous system (CNS) is extremely uncommon. Treatment and outcome data from previously published cases may provide some guidance in light of the ongoing fungal meningitis outbreak in 2023 involving *Fusarium* spp. in the United States and Mexico.

**Methods:**

We reviewed the published literature describing cases of invasive fusariosis of the (CNS) that included data on patient demographic characteristics, treatment, and outcome.

**Results:**

Twenty-six cases met inclusion criteria. The mean age was 36 years, 55% involved females, 60% had underlying hematologic malignancy, and another 16% were on immunosuppressants. The majority of infections were from *Fusarium solani* species complex. Overall 72% of patients died. The majority received monotherapy with amphotericin B, although some received voriconazole monotherapy or combination therapy with amphotericin B plus voriconazole with or without adjuvant surgery. Among the survivors, 3 received amphotericin B monotherapy, 2 voriconazole monotherapy, 1 combination therapy of both, and one surgery only.

**Conclusion:**

The overall mortality rate in published cases of fusariosis of the CNS was high, although—unlike during the current outbreak—the preponderance of patients were severely immunocompromised. While historically the majority were treated with amphotericin B monotherapy, some recent patients were treated with voriconazole monotherapy or combination therapy with amphotericin B plus voriconazole. Current guidelines recommend monotherapy with voriconazole or lipid formulations of amphotericin B or combination of both for the treatment of invasive fusariosis, which is in line with the findings from our literature review and should be considered during the ongoing 2023 outbreak.

## Introduction

*Fusarium* species are filamentous fungi ubiquitous in the environment and rarely cause invasive infection [1]. However, in many countries of the world, fusariosis ranks as the third most prevalent mold infection after aspergillosis and mucormycosis [1–4]. Risk factors for disease include profound or prolonged neutropenia, hematologic malignancy or allogeneic stem cell transplants [1, 5]. Fusariosis typically presents with fungal pneumonia, skin lesions and fungemia, while infections of the central nervous system (CNS) have been very rarely reported [6, 7]. Disseminated fusariosis has a fatality rate of 50–70% in immunocompromised individuals [8].

Despite the relative infrequency of this disease manifestation, an outbreak of *Fusarium* meningitis is currently ongoing [9]. Patients receiving epidural anesthesia in two clinics in Matamoros, Mexico have been identified at risk for *Fusarium* meningitis, with 10 confirmed cases, 9 deaths and another 22 probable or suspected cases in Matamoros involving United States (U.S.) cases, as of July 21, 2023 [10], with additional cases involving persons residing in Mexico. An additional outbreak occurred in late 2022 and early 2023 in Durango, Mexico, involving individuals receiving spinal anesthesia, including obstetric patients, with at least 79 cases of meningitis and 35 deaths with *Fusarium* spp. identified in a relevant proportion of cases as the causative organism [11]. The relationship, if any, between these locations and any potential common source remains unclear. While U.S. and Mexican Health Authorities continue their investigations, it is of paramount importance to review past cases in an attempt to guide current treatment regimens and ascertain prognostic information using prior reports of CNS fusariosis. We therefore reviewed the published literature for cases of CNS fusariosis to analyze treatment and outcome data.

## Methods

We reviewed the published literature describing cases of invasive fusariosis involving the CNS, focusing on demographic characteristics of cases such as age and biologic sex, risk factors of disease such as immunosuppression or malignancy, location of infection, antifungal treatment, and outcome.

We searched PubMed, Google Scholar, and Web of Science using the keywords “Fusarium”, “fusariosis”, “CNS”, “brain”, and “central nervous system “ to select relevant clinical studies and case reports published in English between Jan 1, 1963 and July 20, 2023. We also searched citation lists of all relevant publications for additional references and contacted authors, when available, for additional case information.

Information on individual cases, namely age, sex, underlying diseases, extent of fusariosis, causative pathogen, treatment and outcomes was abstracted from the literature where available. Descriptive analysis was performed.

## Results

Our literature review revealed 27 cases of CNS infections caused by *Fusarium* spp*.*, although one case report was unevaluable as it lacked any clinical data [12]. Thus, this case review included 26 cases in total (Table 1).

**Table 1:** Prior reports of Central Nervous System (CNS) or Meningeal Fusariosis

#/Ref./Year published	Age	Sex	Risk Factors	Causative Species	Extent of Disease	Treatment	Outcome (day of death/follow up)
1[31] / 2022	70	NA	Relapsed AML receiving induction therapy	*Fusarium spp.*	Maxillary sinus with intraorbital extension and meningitis	L-AMB 8 mg/kg + VOR + intrathecal AMB	NA
2[32] / 2022	32	F	Prednisolone, methotrexate, adalimumab, rheumatoid arthritis	*F. proliferatum*	Brain mass with meningo-encephalitis	IV VOR (12 mo) + surgery	Alive (9 months follow-up)
3[33] / 2022	6	M	Tuberculous meningitis	*F. falciforme*	Multiple brain abscesses and meningitis	AMB 5 mg/kg + VOR + TERB + surgery	Deceased (1 month)
4[34] / 2016	14	F	Dexamethasone (4 mg, 3x/day for 14 days), Turberculous meningitis	*Fusarium spp.*	Meningitis	AMB (14 days)	Alive (25 days)
5[13] / 2015	50	F	DM and ALL with failed auto-HSCT, ponatinib and dexamethasone	*F. solani*	Nasal septum ulcer, 1 cm brain mass	VOR + ABLC 5 mg/kg + GCSF + surgery	Hospice/Deceased (1 month)
6[35] / 2014	33	F	None	*Fusarium spp.*	Brain mass	VOR + TER + AMB + surgery	Alive (18 months)
7[36] / 2008	53	F	Promyelocytic leukemia	*F. oxysporum*	Brain abscess	VOR	Alive (5 months)
8[37] / 1998	NA	NA	ALL	*Fusarium spp.*	Brain abscess	surgery	Alive (NA)
9[38] / 1983	17	F	Prednisone (60 mg/day)	*Fusarium spp.*	Brain abscess and meningitis	AMB + intrathecal AMB + surgery (burrhole aspiration)	Deceased (7 days)
10[39] / 1974	2	F	60% burn	*Fusarium spp.*	Brain, skin, kidney	None (autopsy)	Deceased (42 days)
11[40] / 1986	53	M	Multiple myeloma	*F. oxysporum*	Skin, blood, brain	AMB	Deceased (30 days)
12[41] / 1991	15	M	ALL	*Fusarium spp.*	Leptomeningitis	AMB, 5FC, Miconazole + granulocyte transfusion	Deceased (NA)
13[42] / 1996	6	F	Aplastic anemia, allo BMT	*Fusarium spp.*	Skin, lung, brain	AMB	Deceased (NA)
14[43] / 2003	76	M	AML	*F. solani*	Brain abscess	ABLC 5 mg/kg then L-AMB 7.5 mg/kg, then VOR	Deceased but negative for *Fusarium* at autopsy (30 weeks)
15[44] / 2009	55	F	Lung transplant	*Fusarium spp.*	Brain abscess	None (autopsy)	Deceased (less than 7 days; not specified in report)
16[45] / 2007	NA	NA	NA	*F. solani*	Meningitis	AMB	Deceased (NA)
17[46] /2013	23	M	FSGS	*Fusarium spp.*	Brain and detection in blood and urine	None (autopsy)	Deceased (NA)
18[47] / 1997	41	M	AML, allo SCT, GVHD	*Fusarium spp.*	Brain, lung, skin	ABLC	Deceased (8 days)
19[47] / 1997	71	M	AML	*Fusarium spp.*	Brain, sinuses, skin	AMB	Deceased (4 days)
20[47] / 1997	42	M	CLL, allo SCT	*Fusarium spp.*	Brain, sinuses, lung, skin	L-AMB	Partial response / Deceased (85 days)
21[6] / 2003	7	F	aplastic anemia, allo SCT	*Fusarium spp.*	Stroke, lung, skin	AMB	Deceased (5 days)
22[6] / 2003	42	F	AML	*Fusarium spp.*	Cavernous thrombosis, lung, skin	AMB	Alive (NA)
23[6] / 2003	49	M	Multiple Myeloma, allo SCT, GVHD	*F. solani*	Brain, lung, skin	AMB, VOR	Deceased (41 days)
24[6] / 2003	22	M	AML, allo SCT, GVHD	*Fusarium spp.*	Brain, lung, skin	AMB	Deceased (11 days)
25 [48] / 2017	65	F	DM and cirrhosis	*Fusarium spp.*	Brain abscesses, meningitis	AMB then L-AMB	Deceased (3 months)
26 [49] / 2015	NA	NA	Chronic granulomatous disease	*F. falciforme*	Epidural abscess, meningitis, cervical myelitis	NA	Deceased (NA)

ABLC: amphotericin B lipid complex; ALL: acute lymphocytic leukemia; alloSCT: allogeneic stem cell transplant; AMB: amphotericin B; AML: acute myeloid leukemia; BMT: bone marrow transplant; CLL: chronic lymphocytic leukemia; DM: diabetes mellitus; FSGS: focal segmental glomerulosclerosis; GCSF: granulocyte colony stimulating factor; GVHD: graft versus host disease; HSCT: hematopoietic stem cell transplant; L-AMB: liposomal amphotericin B; NA: not available; TERB: terbinafine; VOR: voriconazole

**Fig. 1:**
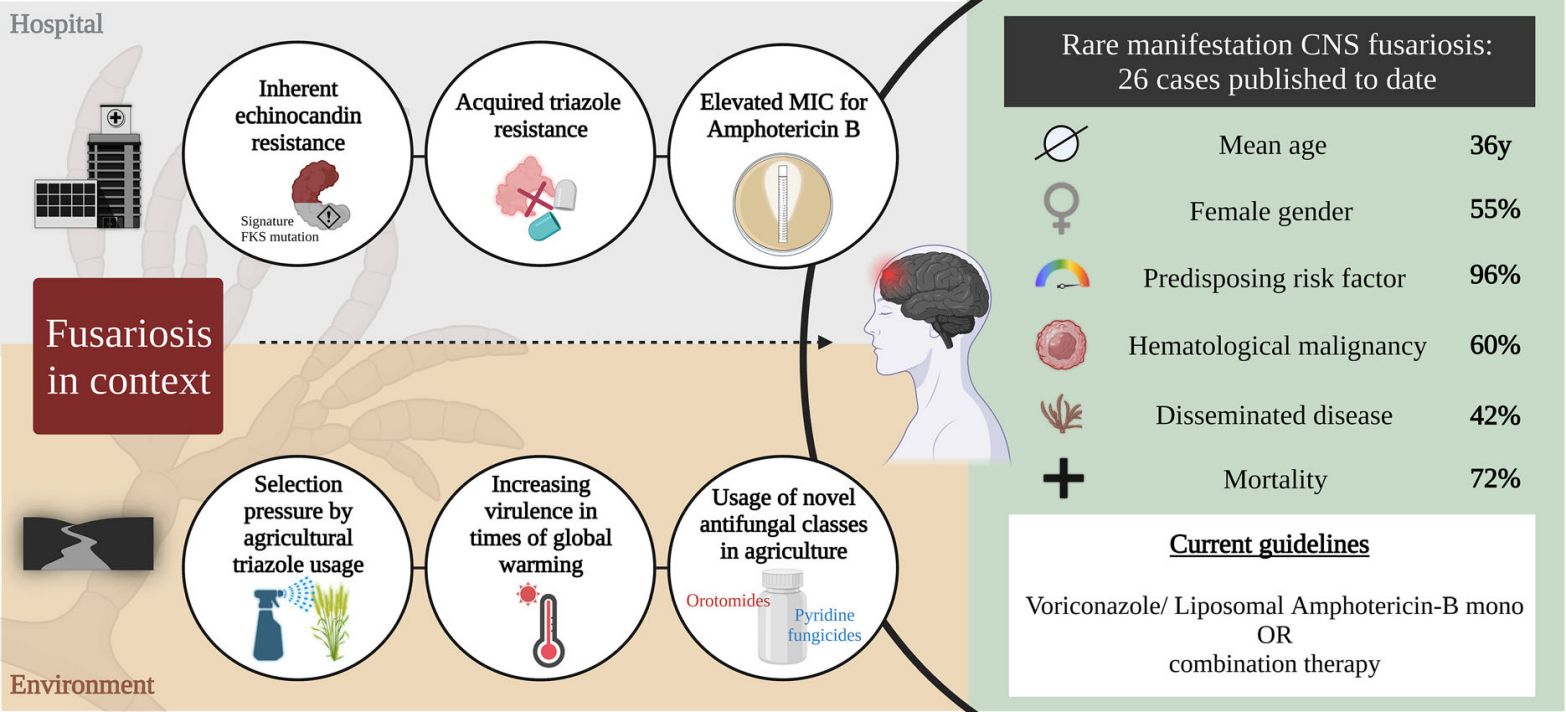
Interchange between fusariosis in the environment and the healthcare setting, and the problem of antifungal resistance. Key Takeaways from this review on CNS fusariosis and treatment recommendation

Mean age of cases with CNS fusariosis was 36 years (range 2–76 years, data available from n = 22), and 12/22 (55%) of cases occurred in females, with the remaining 10/22 cases (45%) occurring in males (data not available in 4 cases). Risk factors (or absence of any risk factors) were reported in 25/26 cases and hematological malignancies/diseases were observed most commonly (15/25 cases, 60%), while another 4 cases received immunosuppressive treatment for other conditions. Of note, in only one case was it reported that patients had no known risk factor for disease (Table 1; Fig. 1).

Clinical manifestations varied widely, including meningitis, leptomeningitis, meningoencephalitis, brain abscesses and masses, cavernous thrombosis and stroke. Of note, 11/26 cases (42%) developed CNS fusariosis as part of their disseminated disease which almost always involved the lungs and skin, with positive blood cultures obtained in a relevant proportion of cases. Of those 11 with disseminated disease including CNS fusariosis, 9/11 (82%) had underlying hematological malignancy. *Fusarium solani* species complex was the causative pathogen in the majority of cases with species identification, while *Fusarium oxysporum* and *Fusarium proliferatum* were also detected. Fungal biomarkers and other diagnostics were rarely utilized; only in one single case report serum galactomannan and beta-D-glucan were tested, both giving negative results [13].

In terms of treatment, 13/21 cases (62%; in three cases CNS fusariosis was only diagnosed at autopsy, one additional case received surgery only and in one no data was available) received amphotericin B, with more recent cases mostly receiving lipid formulations of amphotericin B. Also 10/21 cases occurred before voriconazole was approved for clinical use. In some cases intrathecal amphotericin B was added, or later stepdown to oral voriconazole performed. Five cases (24%) received combination therapy with a polyene plus voriconazole ± additional antifungal therapy (e.g. terbinafine), while two cases (10%) received voriconazole monotherapy (Table 1). Surgery was performed in a total of 6/22 (27%) of cases.

Overall 18/25 cases (72%) had a fatal outcome, while 7/25 (28%) survived (for one case no outcome data available). The survivors included patients without any known risk factors, and four patients with underlying hematological malignancy; three of the survivors received polyene monotherapy (one of them later stepping down to oral voriconazole), two received voriconazole monotherapy, while each one received antifungal combination therapy or surgery only. Information regarding neutrophil count recovery in the hematologic malignancy patients was not available.

## Discussion

Invasive fusariosis, although rare, is one of the most common mold infections, typically affecting immunocompromised persons. While fusariosis is one of the most frequent invasive mold infections, cases involving the CNS have been rarely reported and have nearly exclusively occurred in patients with known risk factors prior to the recent *Fusarium* meningitis outbreak in Durango, Mexico, and the current ongoing outbreak involving *Fusarium solani* species complex among patients who underwent procedures involving epidural anesthesia at two clinics in the city of Matamoros, Tamaulipas, Mexico. *F. solani* has been detected in CSF in patients receiving follow-up care in both Mexico and the U.S. In addition, elevated levels of β-D-glucan have been detected in the CSF of at least 6 of these patients. In total, 212 residents in 25 U.S. states and jurisdictions who underwent epidural anesthesia at these two clinics are deemed to be at risk for *Fusarium* meningitis [14]. Thus, guidance on how best to diagnose and treat these infections is important.

While culture from CSF, CNS abscess formations or blood remains the diagnostic gold standard, fungal biomarker testing was reported in only 1/26 cases reviewed here. However, fungal biomarkers tested from serum and CSF, like galactomannan and β-D-glucan, can be positive in fusariosis [1] and aid in the diagnosis of this CNS invasive fungal infection that carries very high morbidity and mortality [15], as does panfungal PCR from CSF or tissue. Next generation sequencing from blood specimens, which has shown promise for other mold infections [16], may present an important diagnostic method in the future, allowing detection of the causative species.

We reviewed the literature on published cases of fusariosis involving the CNS, and identified a total of 26 cases published between 1974 and 2022. The majority of cases involved young patients with a mean age of 36 years. Fifty-five percent involved females and 60% had underlying hematologic malignancy, with another 16% on immunosuppressants. The majority of infections were caused by *Fusarium solani* species complex. Overall, 72% of patients died. While the majority received monotherapy with amphotericin B (also because a significant proportion of cases occurred before voriconazole was approved for clinical use), most of the newer cases received liposomal amphotericin B or voriconazole monotherapy, with some receiving combination therapy with amphotericin B plus voriconazole with or without adjuvant surgery.

Although the majority of patients involved in the current outbreak in Mexico and the U.S. likely lack many of the risk factors found in the published cases – particularly hematologic malignancy and immunosuppression – treatment and patient outcomes from the published cases reviewed here may be instructive. Among the survivors from the published cases, 3 received amphotericin B monotherapy, 2 voriconazole monotherapy, 1 combination therapy with amphotericin B plus voriconazole, and one surgery only. Treatment with voriconazole, lipid formulations of amphotericin B, or combination therapy with these two agents have been recently recommended as first line options [1]. While no MIC testing was performed in the vast majority of cases reviewed here, it is known that *Fusarium* spp. are intrinsically resistant to echinocandin therapy, and commonly have elevated MICs to both triazoles and polyenes [17, 18]. Past reports, in vitro susceptibility and murine models of infection suggest that combination therapy with liposomal amphotericin B and voriconazole should be used as first-line therapy [19], with the rationale to extend the spectrum of antifungal activity against these highly resistant pathogens. Intrathecal therapy may be used as adjunctive therapy, however consultation with those highly experienced with this treatment are recommended when considered for use. Data is insufficient to inform species-specific antifungal treatment recommendations [1]; although its clinical implications remain unclear [2], MIC testing should therefore be performed and may also guide therapy and novel antifungals obtained through compassionate use programs may also be needed in refractory cases, based on availability [20]. Novel antifungal compounds that are currently in clinical development, namely fosmanogepix or olorofim, have good CNS penetration and could also provide future treatment options for this deadly disease caused by *Fusarium* spp. that often show very high MICs against most available antifungals in vitro. [21–23].

Innate drug resistance in *Fusarium* spp. likely evolved in the environment as a survival mechanism. The presence of efflux pumps in *Fusarium* spp. have been shown to protect against phytoalexins, which are low molecular weight antimicrobial compounds produced by plants as a response to biotic and abiotic stresses, and the triazole fungicide tebuconazole [24, 25]. Thermotolerance in times of global warming may further contribute to increased virulence in some *Fusarium* spp., as shown for *Fusarium graminearum* species complex, a pathogen to wheat and other cereal crops which can result in yield losses up to 75%, particularly during warmer and humid years [26]. This increased virulence leading to increased threat of food supply may lead to increased usage of fungicides in agriculture, including not only azoles like tebuconazole, but because of increasing resistance also fungicides with new mode of action like iplufenoquin which shares its mode of action with a new antifungal olorofim [27], or the fungicide aminopyrifen, which shares its mode of action with fosmanogepix [28]. This broad use of fungicides with similar mechanisms of actions with the antifungals used to treat infections in humans may lead to a further increase in antifungal resistance in the future (Fig. 1).

The relative lack of fusariosis cases in immunocompetent hosts speaks to the opportunistic nature of this genus. However, the attack rate and accompanying morbidity and mortality of infection in those receiving spinal anesthesia as the source reminds us that virulence cannot be insinuated for all pathogens. Under the right circumstances, it is likely that most microbes have the potential for virulence under certain conditions [29]. For example, *Exserohilum* was not a known cause of human CNS infections until a fungal meningitis outbreak with this organism was described in 2013 [30].

Of note, the current fusariosis outbreak in Matamoros, Mexico involved medical tourism. The majority of those affected were from the U.S. and underwent procedures in Mexico that had shorter waiting times and were lower cost. The high cost of health care for many in the U.S. is undoubtedly linked to these two recent outbreaks, and individuals seeking low-cost health care abroad may continue to be at risk for poor outcomes in the future.

In conclusion, two recent outbreaks of fusariosis involving the CNS, including one that is ongoing in Matamoros, Mexico and the U.S., are related to receipt of epidural anesthesia in Mexico. It is unclear if these two outbreaks are related. Given the high morbidity and mortality associated with these cases of invasive fusariosis, it is important to understand which treatment regimens may be best to treat these infections. Current guidelines recommend first-line treatment with voriconazole monotherapy, lipid formulations of amphotericin B, or combination therapy with these two regimens [20]. This case series also found that the majority of survivors from fusariosis involving the CNS were treated with voriconazole or amphotericin B monotherapy, with an additional patient treated with combination therapy using these agents and another from surgery alone. The findings in this case series and current guidelines for the treatment of invasive fusariosis should be instructive in the treatment of these patients.
